# Angiography-Based Fractional Flow Reserve: State of the Art

**DOI:** 10.1007/s11886-022-01687-4

**Published:** 2022-04-18

**Authors:** Alessandra Scoccia, Mariusz Tomaniak, Tara Neleman, Frederik T. W. Groenland, Annemieke C. Ziedses des Plantes, Joost Daemen

**Affiliations:** 1 Department of Cardiology, Thoraxcenter, Erasmus University Medical Center, room Rg-628, P.O. Box 2040, 3000 CA Rotterdam, The Netherlands; 2 First Department of Cardiology, Medical University of Warsaw, Warsaw, Poland

**Keywords:** Angiography-based FFR, Percutaneous coronary intervention, Functional lesion assessment, Quantitative flow ratio, Vessel FFR, FFR_angio_

## Abstract

**Purpose of Review:**

Three-dimensional quantitative coronary angiography-based methods of fractional flow reserve (FFR) derivation have emerged as an appealing alternative to conventional pressure-wire-based physiological lesion assessment and have the potential to further extend the use of physiology in general. Here, we summarize the current evidence related to angiography-based FFR and perspectives on future developments.

**Recent Findings:**

Growing evidence suggests good diagnostic performance of angiography-based FFR measurements, both in chronic and acute coronary syndromes, as well as in specific lesion subsets, such as long and calcified lesions, left main coronary stenosis, and bifurcations. More recently, promising results on the superiority of angiography-based FFR as compared to angiography-guided PCI have been published.

**Summary:**

Currently available angiography -FFR indices proved to be an excellent alternative to invasive pressure wire-based FFR. Dedicated prospective outcome data comparing these indices to routine guideline recommended PCI including the use of FFR are eagerly awaited.

## Introduction

A significant body of evidence has demonstrated that coronary revascularization should be tailored to ischemia, rather than anatomy, in order to improve symptoms and prognosis [1–3]. Physiological lesion assessment using either fractional flow reserve (FFR) or instantaneous wave-free ratio (iFR) is the current guideline-recommended invasive gold standard for assessing the ischemic potential of an angiographically intermediate coronary stenosis [4, 5]. However, despite the robust data, the uptake of FFR in routine clinical practice remains low, reportedly due to need for hyperemia associated with patient discomfort, additional pressure wire instrumentation, and presumed additional time and costs related to invasive physiological lesion assessment under hyperemic conditions [6, 7]. In order to overcome some of the aforementioned limitations, alternative methods, including non-hyperemic pressure ratios (NHPR) and angiography-based FFR, have been proposed. Among angiography-based methods, 2-dimensional quantitative coronary angiography (2D QCA) was the first to be commercially available, however demonstrated to have only a modest correlation with physiologic indices of ischemia, such as FFR [8–10]. Conversely, 3D QCA proved to have a higher accuracy and a stronger correlation with FFR as compared with 2D QCA by reducing the effects of fore-shortening and non-symmetric coronary lesions [11–14]. Improved understanding of the correlations between QCA and pressure flow measurement, advances in computational power, simplified computational fluid dynamics or flow resistance analysis, and 3D coronary angiography reconstructions have made the development of angiography-based FFR methods possible [15–17].

### The Concept of Simplified Computational Fluid Dynamics

Computational fluid dynamics (CFD) is the most widely used method to solve the equations which describe the motion of fluids, the Navier–Stokes equations. The solution of these equations provides information about velocity and pressure at any location in the coronary artery at any point of time. Since the solution of Navier–Stokes equations can be time consuming and computationally expensive, simplified analyses using equations built on the seminal work of Young, Tsai, and Gould have been proposed [18, 19].

The difficulty in virtual estimation of pressure drop arises from the fact that hyperaemic flow, as required for FFR assessment, is variable and difficult to quantify for each specific artery, since flow intrinsically depends on the vasodilation of the underlying myocardium and the haemodynamic status of the patient [20, 21].

However, the technology is quickly gaining momentum and several methods for FFR computation, combining the simplified CFD model with 3D QCA from invasive angiography, have been proposed.

### Currently Available Angiography-Based FFR Solutions

**Fig. 1:**
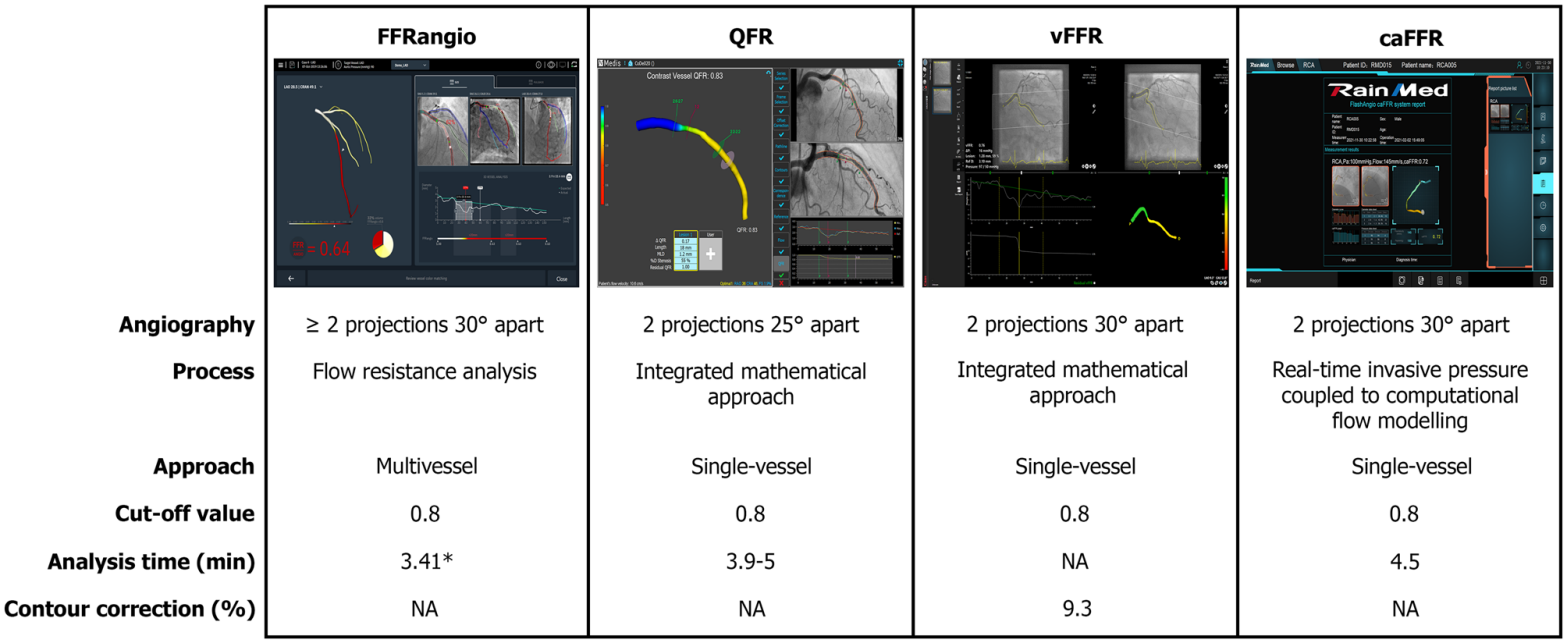
Commercially available software for angiography-based FFR. *Data on file, unpublished data provided by CathWorks. (Photo permissions: FFRangio with permission from CathWorks; QFR with permission from Medis Medical Imaging Systems B.V.; vFFR with permission from Pie Medical Imaging B.V.; and caFFR with permission from RainMed Medical Technology Co., Ltd.)

To date, four angiography-based FFR methods have emerged and are currently commercially available (Fig. 1):

- FFR_angio_ is a resting, adenosine-free angiography-based index, developed by CathWorks, Ltd, Kfar Saba, Israel. Using two or more angiographic projections at least 30° apart, the software provides a 3D reconstruction of the entire coronary tree, modeled as an electric circuit with each segment acting as a resistor, according to its length and diameter. For the hemodynamic evaluation, the contribution of each narrowing to the total flow resistance is taken into account based on its impact on overall resistance and a subsequent lumped model is built, allowing the pressure drops and the flow rates to be estimated. Two models of the coronary tree are built, a first model with stenosis, and a second model without stenosis. FFR_angio_ is subsequently calculated as the ratio of maximal flow in the presence and absence of stenotic lesions [22, 23].
- Quantitative flow ratio (QFR) developed by Medis Medical Imaging System, Leiden, the Netherlands, and Pulse Medical Imaging Technology, Shanghai, China, is obtained from two diagnostic angiographic projections, at least 25° apart to generate a 3D QCA model. In the QFR model, the pressure drop is calculated for each segment using the stenosis geometry and mean hyperemic flow velocity, using the Gould formula [16]. Blood is treated as a homogeneous and Newtonian fluid, coronary pressure is assumed to be constant in the absence of stenosis, coronary flow velocity to be preserved along the coronary, and steady flow is specified as boundary condition at the outlet. Hence, the mass flow rate at each location along the interrogated vessel can be determined by the mean flow velocity and the vessel sizing from 3D QCA [13, 24]. In the FAVOR pilot study, the computation of the hyperemic flow was initially obtained and tested based on a per vessel basis using 3 different flow models: (1) a fixed empiric hyperemic flow velocity (fixed-flow QFR (fQFR)); (2) modeled hyperemic flow velocity on the basis of the TIMI frame count analysis without pharmacologically induced hyperemia (contrast-flow QFR (cQFR)), and (3) measured hyperemic flow velocity derived from coronary angiography during adenosine-induced maximum hyperemia (adenosine-flow QFR (aQFR)) [24]. The authors observed good agreement with FFR for all the measurements (fQFR 0.003 ± 0.068, cQFR 0.001 ± 0.059, and aQFR 0.001 ± 0.065) [24]. The diagnostic accuracy of cQFR ≤ 0.80 for predicting FFR less than or equal to 0.80 was higher than that of fQFR ≤ 0.80 and comparable with that of aQFR ≤ 0.80. Therefore, cQFR is the currently used model [24]. Comparative computation for QFR vs FFR was reported in FAVOR II Europe Japan indicating a significantly shorter time for QFR computation (5.0 vs 7.0 min, *p* < 0.001). In the FAVOR III China study, QFR computation required 3.9 ± 1.4 min [25, 26••].
- Vessel fractional flow reserve (vFFR) developed by CAAS, Pie Medical Imaging, Maastricht, the Netherlands is obtained from two angiographic views with at least 30° difference in rotation/angulation to generate the 3D QCA. Within the CAAS workstation, CFD approach models flow using a simplified Navier–Stokes equation, applying boundary conditions as a constant parabolic flow profile at the inlet and a stress-free outlet, a rigid-wall, non-slip conditions, and a Newtonian fluid approximation of blood. The pressure drop is calculated by applying physical laws including viscous resistance and separation loss effects present in coronary flow behavior, as described by Gould and Kirkeeide et al. [27]. Maximum hyperaemic blood flow was empirically determined from clinical data and assumes that proximal coronary velocity is preserved along the coronary artery [27]. vFFR is computed automatically, using the invasively measured aortic root pressure as an input boundary condition [27]. The algorithm applies automated and harmonized optimal end-diastolic frame selection in the two orthogonal projections by ECG triggering and allows physiological lesion assessment of a specific target segment or vessel of interest, precluding the need to perform an assessment of the full cardiac tree or manual frame counting [27].
- Computational pressure-flow dynamics derived FFR (caFFR) developed by Rainmed Ltd, Suzhou, China is a technique based on the 3D reconstruction of the vessel from two angiographic projections at different angles (≥ 30°). The resting coronary flow velocity is determined using the TIMI frame count while the aortic pressure is recorded by the FlashPressure pressure transducer connected to the guide catheter and transmitted to FlashAngio console, which automatically determines mean aortic pressure over the third to eighth cycles following angiography [28]. The flow velocity and the mean aortic pressure are used as an input in the FlashAngio software which calculates the pressure drop along the generated mesh of the coronary artery. Compared to the previous software, caFFR uses a real time invasive pressure coupled to computation flow modeling to determine the pressure drop across a stenosis. This allows to take into account the dynamic nature of blood pressure, instead of using a static value of aortic pressure, and to account for energy loss in lumen area proximal and distal to the stenosis. The data are further processed with a CFD technique that provides the characteristics of intravascular blood flow and the pressure field, enabling the computation of the pressure gradient between the inlet and outlet of the studied coronary segment [28]. Time to computation was highlighted in FLASH FFR, showing that caFFR analysis required a total operation time of less than 5 min with less than 1 min computation time [28].

### Interobserver Variability

A common feature of all angiography-based FFR software is the need for specific user interaction to refine geometrical vessel parameters and to select appropriate angiographic projections and frame. These changes performed by individual operators may affect the final physiological result and might have an impact on the reproducibility of the technology. However, data about time and amount of necessary manual contour corrections are provided only for vFFR, which showed a highly accurate contour detection, and a percentage of manual contouring correction needed in only 9.3% of vessels [29].With respect to interobserver variability, vFFR proved to have a low interobserver variability when performed either by experienced operators (*r* = 0.95), or when performed by a blinded CoreLab or independent trained local site personnel [27]. In the recently published FAST II study, a high reproducibility between CoreLab and on-site measurement (*r* = 0.87) was demonstrated, consistent among specific lesion and patient subsets [29]. In this regard, FFR_angio_ has shown good reproducibility (*r* = 0.88) when performed offline by experienced operators; however, data about the agreement of on-site operators reproducibility are lacking [22, 30]. QFR showed a good reproducibility when computed by two independent CoreLabs (*r* = 0.96) or when performed online versus an independent CoreLab (*r* = 0.91) [31, 32]. However, the recently published QREP study demonstrated a modest reproducibility of QFR when computed by multiple observers, dependent on stenosis severity, angiographic quality, and the observer [33].

## Clinical Validation Studies

### FFR_angio_

FFR_angio_ showed a high diagnostic accuracy when compared to FFR as a reference in small exploratory studies, as well as in a larger validation study when performed offline by experienced operators [23, 30, 34] (Table 1). The multicenter, prospective observational FAST-FFR study confirmed a high diagnostic performance of FFR_angio_ when computed online by trained local site personnel (sensitivity 94%, specificity 91%, and diagnostic accuracy 92%) that remains high when only considering FFR values between 0.75 and 0.85 (diagnostic accuracy 87%) [22]. A pooled analysis of five prospective cohort studies reported an excellent diagnostic performance in a large cohort of patients, which was consistent in the overall cohort (sensitivity 91%, specificity 94%, and diagnostic accuracy 93%) and across patients (including age, sex, BMI, diabetes, clinical presentation, and lesion types) [35]. The diagnostic performance was confirmed in patients with multivessel disease and when compared to FFR in reclassification of coronary disease severity according to SYNTAX score [36]. In a post hoc analysis of FAST-FFR, FFR_angio_ showed a high diagnostic performance independent of most patient characteristic, though its specificity varied according to the vessel (98.7% for LAD, 86.3% for LCx, and 84.3% for RCA; *p* = 0.046) [37]. Interestingly, data regarding clinical outcome in a real-world population have been recently published, showing a low one year rate of MACE in patients where the treatment decision was based on the FFRangio results (4.1% and 2.5% for the revascularization and deferral groups, respectively) [38•].

**Table 1:** Major studies investigating the diagnostic performance of Pre-PCI angiography-based fractional flow reserve

**Study/author**	**Software**	**Year**	**Study design**	**Number of vessel (patient)**	**AUC**		**Sensitivity %**	**Specificity %**	**PPV %**	**NPV %**	**Accuracy %**	**Annotations**
**Core lab/in-procedure measurment**												
Kornowski et al	FFR_angio_	2016	Prospective	101 (88)		88		98			94	
Trobs et al	FFR_angio_	2016	Retrospective	100 (73)	0.93	79		94	85	92	90	
Pellicano et al	FFR_angio_	2017	Prospective	203 (184)		88		95			93	
FAST FFR	FFR_angio_	2019	Prospective	319 (301)	94	94		91	89	95	92	
Omori et al	FFR_angio_	2019	Prospective	118 (50)		92		92			92	
FAVOR pilot study	cQFR	2016	Prospective	84 (73)	0.92	74		91	80	88	86	
FAVOR II China study	QFR	2017	Prospective	332 (308)	0.96	95		92	86	97	93	
Yazaki—Ishii	QFR	2017	Retrospective	151 (142)	0.93	89		89	77	95	89	
The WIFI II study	QFR	2018	Substudy	292 (191)	86	77		86	75	87		
The FAVOR II Europe-Japan	QFR	2018	Prospective	317 (329)	0.92	87		87	76	93	87	
Choi et al	QFR	2020	Registry	599 (452)	0.95	92		91	87	95	91	
Westra et al	QFR	2019	Meta-analysis	969 (819)		84		88	80	95	87	
Zuo et al	QFR	2019	Meta-analysis	8213	0.92	90		88				
FAST study	vFFR	2019	Retrospective	100 (100)	0.93							
FAST EXTEND	vFFR	2020	Retrospective	294 (294)	0.94	75		94	84	89	88	
FAST II	vFFR	2021	Prospective	500 (334)	0.93	81		95	90	90	90	
FLASH FFR	caFFR	2019	Prospective	328	0.98	90		99	97	95	95.7	
**Microvascular dysfunction**												
Ai et al	caFFR	2020	Retrospective	57 (56)	0.92	86		81	89	77	84	Against IMR 25
Mejia Renteria et al	QFR	2018	Substudy	115 (104)							IMR < 23 = 88%, IMR ≥ = 76%	
**Diabetes**												
Smit et al	QFR	2019	Prospective	320 (259)		71–69		95–91	85–74	89–88	88–85	
**Previous MI**												
Emori et al	QFR	2018	Retrospective	200 (150)							87 vs 92	
**In stent restenosis**												
Tang et al	QFR	2021	Retrospective	185 (177)								QFR ≤ 0.94 predictors of VOCE
Cai et al	QFR	2021	Retrospective	226 (208)								QFR ≤ 0.94 predictors of ISR
**Severe aortic stenosis**												
Renteira et al	QFR	2020	Retrospective	138 (115)	0.93–0.97						88	
Kleczynski et al	QFR	2021		416 (221)								
**Non culprit lesions in ACS**												
Hansen et al	QFR	2019	Post hoc	146 (118)	0.89	92		94	94	91		
Lauri et al	QFR	2020	Retrospective	91 (88)	0.91	86		80	78	87	84	
Tebaldi et al	QFR	2020	Prospective	184 (116)	0.96	72		94	81	90	88	
Bar et al	QFR	2021	Post hoc	946 (617)								
Milzi	QFR	2021	Retrospective	280 (220)	0.89	84		86				
**Angiography-based FFR vs iFR**												
Watarai et al	QFR	2019	Prospective	150	0.91	85		83	72	91		
Kleczyński et al	QFR	2021	Meta-analysis	110	0.87	76		83			80	
Hwang et al	QFR	2019	Retrospective	253 (182)		92		90	86	95	91	

Abbreviations: *ACS* acute coronary syndrome, *AUC* area under the curve, *caFFR* computational pressure-flow dynamics derived FFR, *FFR* fractional flow reserve, *iFR* instantaneous wave free ratio, *MI* myocardial infarction, *NPV* negative predictive value, *PPV* positive predictive value, *QFR* quantitative flow ratio, *vFFR* vessel fractional flow reserve

Finally, in a head-to-head comparison between NHPR and FFR_angio_ in predicting FFR, FFR_angio_ agreed more often with invasive FFR than NHPRs [39].

### Quantitative Flow Ratio (QFR)

Quantitative flow ratio (QFR) is currently the angiography-based index with the largest amount of evidence. It was first validated in the FAVOR pilot study which assessed the superiority of different QFR approaches (fQFR, cQFR, and aQFR), computed offline, over 3D-QCA in predicting FFR (diagnostic accuracy of 80%, 86%, and 87% vs 65%, respectively) [24].

Two multi center studies, the FAVOR II Europe-Japan and FAVOR II China evaluated the feasibility and the diagnostic performance of online QFR, demonstrating a high agreement between QFR and FFR (mean difference: −0.01 ± 0.06, in both studies) [25, 40]. In the FAVOR II China study, the diagnostic accuracy of QFR on a vessel- and patient-level was 92.7% and 92.4% respectively, while in FAVOR II Europe Japan, the diagnostic accuracy of computation of QFR was 86.8% [25, 40].

Offline and online QFR presented high diagnostic accuracy ranging from 83 to 93% and good correlation compared with the gold-standard FFR, consistent among several prospective, and retrospective studies as well as meta-analyses [32, 41–43] (Table 1). Furthermore, its high diagnostic performance was confirmed in large real-world cohorts, showing a superior diagnostic accuracy in predicting FFR positive lesions as compared with resting Pd/Pa ratio (area under the curve (AUC) 0.86; 95% confidence interval (CI): 0.83–0.89 for QFR vs 0.76; 95% CI: 0.72–0.83 for Pd/Pa; *p* < 0.001) [44]. Studies looking at the diagnostic value of QFR with iFR as index reference showed a good correlation and diagnostic performance (*r* = 0.74, AUC 0.91) [45]. Moreover, when using both FFR and iFR as reference standard, QFR correlated better to FFR as compared to iFR (*r* = 0.86 with FFR vs 0.74 with iFR, *p* < 0.001, AUC 0.95 vs 0.88, *p* < 0.001) [46].

Pooled data focusing on the diagnostic performance of QFR and iFR with FFR as a reference, demonstrated that QFR has a higher sensitivity and specificity than iFR (sensitivity 90% vs 79% and specificity 88% vs 85%, respectively, *p* < 0.001 for both) in predicting FFR [47].

Recently, there has been increasing interest in using pre-PCI QFR virtual pullbacks in order to define the pattern of coronary artery disease, either focal or diffuse, and to predict post-PCI functional results. In this context, QFR proved to correctly define the physiological pattern of disease in 83.9% and 91.0% of cases using iFR and FFR pullbacks as reference, respectively [48]. Moreover, based on data from QFR computation, some angiography-derived indexes to assess microcirculatory resistance have been validated and proved to have a good diagnostic performance, as compared to wire-based IMR, both in chronic and acute coronary syndromes (AUC 0.93 and 0.96) [49, 50].

Finally, the multicenter, blinded, randomized FAVOR III China was the first large head-to-head outcome study to be presented. The study demonstrated better clinical outcomes of QFR-guided PCI as compared with angiography-guided PCI at 1 year follow-up. Major adverse cardiac events (MACE) occurred in 5.8% of the patients in the QFR-guided group vs 8.8% in the angiography-guided group (*p* < 0.001), mainly driven by a reduced rate of periprocedural myocardial infarction (MI) in the QFR arm (due to a higher number of lesions deferred for revascularization), reduced rate of MI during follow-up, and lower rates of ischemia driven revascularization [26••]. Moreover, QFR-guided PCI led to a shorter procedure time (53.7 vs 59.4 min, *p* < 0.001), reduced use of contrast media, and lower number of stents implanted [26••]. Of note, the remarkably low number of patients in which no PCI was performed (< 10%) attests to the selection of cases with more severe lesions as compared to previous physiology studies in which only about 50% of patients underwent revascularization [26••, 51]. Longer follow up and the results of the ongoing FAVOR III Europe Japan trial (NCT03729739), assessing whether QFR-based diagnostic strategy yields non-inferior clinical outcome compared to an FFR-based strategy, are eagerly awaited.

### Vessel Fractional Flow Reserve (vFFR)

vFFR was first validated in the retrospective FAST I study (*n* = 100), showing a high diagnostic accuracy when compared to FFR, and was subsequently evaluated in a larger and more heterogeneous cohort of patients, confirming an excellent diagnostic performance among different vessel and anatomy subsets [27, 52] (Table 1).

These positive findings were subsequently confirmed in the prospective, international, and multicenter FAST II study which demonstrated a good correlation between vFFR as calculated by a blinded CoreLab and pressure wire-based FFR (*r* = 0.74; *p* < 0.001; mean bias 0.0029 ± 0.0642) and an excellent diagnostic accuracy of vFFR in identifying lesions with an invasive wire-based FFR ≤ 0.80 (AUC 0.93; 95% CI: 0.90–0.96; *p* < 0.001) also in more complex lesions, including bifurcations, tortuous, and calcified lesions [29].

Interestingly, in a dedicated study focusing on patients with left main coronary artery (LMCA) disease with good quality angiographic visualization and availability of intravascular ultrasound (IVUS) imaging data, 3D-QCA-based vFFR assessment of LMCA disease was shown to correlate well to LMCA minimal lumen area (MLA) as assessed by IVUS (*r* = 0.79, *p* = 0.001). A good diagnostic accuracy of vFFR ≤ 0.80 in identifying lesions with MLA < 6.0 mm2 (sensitivity 98%, specificity 71.4%, AUC 0.95; 95% CI: 0.89–1.00, *p* = 0.001) was observed [53]. Moreover, vFFR computations in patients discussed within the Heart Team in whom the treatment decision was based on angiography alone indicated a considerable proportion (almost one third of the patients) were identified to present with vFFR confirmed lesion significance – revascularization discordance (Tomaniak et al. presented at EuroPCR 2019). The safety and efficacy of a vFFR as compared to an FFR guided revascularization strategy will be assessed in in ongoing multicenter, randomized FAST III trial (NCT04931771).

### Computational Pressure-Flow Dynamics Derived FFR (caFFR)

caFFR was validated in the prospective, multicenter FLASH FFR study, and showed a high correlation and diagnostic accuracy as compared with FFR (*r* = 0.89, diagnostic accuracy 96%; 95% CI: 0.93–0.98), when computed by experienced operators in a low risk patients cohort, although evidence about the accuracy in complex lesions is still lacking [28]. Interestingly, an algorithm for the assessment of microvascular disease, the so called coronary angiography-derived index of microvascular resistance (caIMR) has been assessed in a small cohort of patients with angina and no obstructive coronary artery disease [54]. caIMR proved to be feasible, with a good correlation and diagnostic performance as compared to wire-based IMR (*r* = 0.75, diagnostic accuracy 84%; 95% CI: 72–0.93% and AUC 0.92) [54].

### Gray Zone or Binary Cut-Off

The overall diagnostic performance of angiography-based FFR in identifying FFR-based functional stenosis severity is good. However, the diagnostic accuracy may drop in around 30 to 40% of cases, when angiography-based FFR values are close to the cut-off (0.80), as demonstrated for QFR values between 0.75 and 0.85 (AUC 0.63; 95% CI: 0.42–0.84) [25, 52, 55].

Therefore, the idea of using a “gray zone,” in which FFR assessment could be used to establish stenosis severity, has been proposed. In the FAST EXTEND study, a vFFR gray zone of 0.80 to 0.85 was found in order to have 96% diagnostic accuracy, while in FAVOR II Europe-Japan a gray zone of 0.78 to 0.86 for QFR was defined, with similar diagnostic accuracy [25, 52]. As of to date, no details on the so called gray zone have been presented for both FFR_angio_ and caFFR.

Following previous discussions on the use of a gray zone with iFR, the concept was abandoned and future research continued by using a binary cut-off. As such in FAVOR III China, FAVOR III EU-JAPAN, and FAST III, abandoned binary cut-off for lesions significance of ≤ 0.80 was used. Future studies are needed to better understand and explore the relevance of discordance between angiography-based FFR and FFR, whereas the clinical value of angiography-based FFR using a binary cut-off is currently being studied in several large clinical outcome trials (FAVOR III Europe Japan trial NCT0372973, FAST III NCT04931771) [26••, 29].

## Post-PCI Physiological Assessment

Several studies demonstrated that low post-PCI FFR is linked to higher rates of target vessel failure [56–65]. As such, post-PCI FFR has the potential of acting as a useful tool for the assessment of acute PCI results and might identify cases in need for additional procedural optimization [66, 67]. Despite these observations, post-PCI FFR is still rarely performed in the routine cathlab practice.

Consequently, the diagnostic option of wire-free post-PCI physiological analysis using angiography-based FFR to identify individuals requiring additional diagnostics (IVUS/OCT) and subsequent specific management (i.e., additional stent, post-dilatation) implies options for future procedural improvements.

The FAST POST was the first study to validate vFFR against microcatheter-based FFR in a post-PCI setting, demonstrating a good correlation between conventional invasive post-PCI FFR and vFFR and a high diagnostic accuracy to identify a conventional post-PCI FFR < 0.90 (Table 2) [68].

**Table 2:** Summary of the studies investigating the impact of post-PCI angiography-based fractional flow reserve

**Study/author**	**Software**	**Year**	**Study design**	**Number of vessel (patient)**	**AUC**		**Sensitivity %**	**Specificity %**	**PPV %**	**NPV %**	**Annotations**
**Core lab/in-procedure measurement**											
HAWKEYE	QFR	2019	Prospective	(602)	0.77*	60*		87*			*To predict 2-year VOCE. Cutoff ≤ 0.89
Kogame et al	QFR	2019	Retrospective	(440)	0.70*	65*		64*			*To predict 2-year VOCE. Cutoff ≤ 0.91
FAST POST	vFFR	2021	Retrospective	100 (100)	0.98	80		97	94	88	To predict FFR values < 0.90

Abbreviations: *ACS* acute coronary syndrome, *AUC* area under the curve, *caFFR* computational pressure-flow dynamics derived FFR, *FFR* fractional flow reserve, *iFR* instantaneous wave free ratio, *MI* myocardial infarction, *NPV* negative predictive value, *PPV* positive predictive value, *QFR* quantitative flow ratio, *vFFR* vessel fractional flow reserve, *VOCE* vessel-oriented composite endpoint

*To predict 2-year VOCE

Subsequent data from the HAWKEYE study demonstrated that post-PCI QFR proved to be directly correlated to the risk of future adverse cardiac events (Table 2) [69•]. The vessel-oriented composite endpoint (vessel-related cardiac death, vessel-related myocardial infarction, and target vessel revascularization) was found to be threefold higher in cases with a post-PCI QFR was ≤ 0.89. Consistent observations were also reported at the Transcatheter Cardiovascular Therapeutics 2019 (TCT 2019) for post-PCI vFFR, with vessels presenting post-PCI vFFR values > 0.90 having lower risk of target vessel revascularization at 1 year, as compared to vessels with post-PCI vFFR ≤ 0.90 (1.8% vs. 4.2%, *p* < 0.05) as well as in work performed by Kogame et al. (Table 2) [70, 71].

## Concept of ‘Virtual Stenting’ and Residual Angiography-Based FFR

Recently studies by Biscaglia et al. and Shin et al. have suggested that functional patterns of coronary artery disease categorized as focal, serial, or diffuse based on pre-PCI QFR analyses can predict post-PCI QFR [72, 73]. Using pre-PCI virtual pull backs of QFR, physiological distribution was determined in patients who underwent angiographically successful PCI and post-PCI FFR measurement by pull back pressure gradient index to define predominant focal versus diffuse disease. Interestingly, cumulative incidence of TVF after PCI was significantly higher in patients with predominant diffuse disease [73].

Indeed, the ability to predict the functional outcomes of PCI may constitute another step forward in optimization of PCI results [74]. Recent developments in the 3D-QCA-based FFR software allowed to simulate the effect of ‘virtual’ PCI and estimate post-PCI FFR, termed residual FFR [75]. As of to date, the diagnostic performance of residual QFR and vFFR assessment using baseline angiograms has been evaluated showing a good correlation between invasive post-PCI FFR and post-PCI QFR or vFFR values, respectively, and a good discriminative ability for post-PCI FFR < 0.90 [75, 76]. Such developments have the potential to identify patients expected to have a proper functional PCI outcome as well as those with a lower likelihood of functionally satisfactory outcome, and thus, optimize the treatment and avoid a risk of a futile invasive procedure. Nevertheless, it has to be emphasized that current-generation virtual stenting QFR or vFFR assume an almost perfect PCI result, and thus, cannot account for, i.e., heavy calcifications or stent underexpansion.

## Specific Clinical Settings: Prior MI, Severe Aortic Stenosis, IN-Stent Restenosis

The performance of angiography-based FFR has been evaluated in specific clinical scenarios (Table 1). In patients with prior MI, QFR showed a good correlation with FFR overall, but its diagnostic accuracy was numerically reduced in prior-MI-related coronary arteries compared to non-prior-MI-related coronary arteries (diagnostic accuracy 87% vs. 92%, *p* 0.29) [77]. In the acute setting of STEMI and NSTEMI, QFR measurement in non-culprit vessels appeared to be feasible, reliable, and showed a good diagnostic performance compared to QFR itself and FFR performed in a staged procedure [78–83]. QFR proved also to have an equivalent diagnostic efficiency in assessing functional relevance of in-stent restenosis as in de novo stenosis, though it did not appear a useful tool in predicting in-stent late lumen loss [84, 85].

In patients with severe aortic stenosis, the value of pressure wire-based physiological lesion assessment is still debated given the known attenuation hyperemic response due to increased left ventricle end diastolic pressures and microvascular resistance. However, seminal studies on pre-transcatheter aortic valve implantation (TAVI) QFR showed a good performance compared to post-TAVI FFR as reference [86].

## Conclusion

Angiography-based FFR is emerging as an appealing alternative to conventional pressure-wire physiological lesion assessment and has the potential to further extend the uptake of physiology guided PCI. Whereas promising data have been recently released on the superiority of QFR vs. angiography guided PCI in a Chinese setting, more data is needed to extrapolate these findings to Western populations and guideline recommended invasive physiology PCI as a reference.

As such, the results of currently ongoing dedicated randomized outcome trials are eagerly awaited (FAVOR III Europe Japan trial NCT0372973, FAST III NCT04931771) to fuel discussions with respect to guideline uptake and reimbursement models.

## Abbreviations

aQFR: Adenosine-flow quantitative flow ratio
AUC: Area under the curve
BMI: Body mass index
caFFR: Computational pressure-flow dynamics derived FFR
caIMR: Coronary angiography-derived index of microvascular resistance
CFD: Computational fluid dynamics
CI: Confidence interval
cQFR: Contrast-flow quantitative flow ratio
FFR: Fractional flow reserve
FFR_angio_: Angio-based fractional flow reserve
fQFR: Fixed-flow quantitative flow ratio
iFR: Instantaneous wave-free ratio
IVUS: Intravascular ultrasound
LAD: Left anterior descending
LCx: Left circumflex
LMCA: Left main coronary artery
MACE: Major adverse cardiac events
MI: Myocardial infraction
MLA: Minimal lumen area
NHPR: Non-hyperemic pressure ratios
NSTEMI: Non ST-elevation myocardial infarction
OCT: Optical coherence tomography
PCI: Percutaneous coronary intervention
Pd/Pa: Distal coronary artery pressure to aortic pressure ratio
QFR: Quantitative flow ratio
RCA: Right coronary artery
STEMI: ST-elevation myocardial infarction
vFFR: Vessel fractional flow reserve
2D QCA: 2-Dimensional quantitative coronary angiography
3D QCA: 3-Dimensional quantitative coronary angiography
